# Deciphering the Transcriptomic Dynamics of Self-Incompatibility in Yellow Passion Fruit: Evidence of Modified Sporophytic Mechanism

**DOI:** 10.3390/plants15101564

**Published:** 2026-05-20

**Authors:** Xiaomei Wang, Junzhang Li, Kaichuang Liu, Youmei Huang, Chang An, Yan Cheng, Ping Zheng, Maokai Yan, Biao Deng, Gaifeng Chai, Xiaoping Niu, Hanyang Cai, Yuming Lu, Yuan Qin, Lulu Wang

**Affiliations:** 1 Fujian Provincial Key Laboratory of Haixia Plant Systems Biology, College of Life Science, Fujian Agriculture and Forestry University, Fuzhou 350002, China; wangxiaomei159@163.com (X.W.); kaichuangliu_w@163.com (K.L.); hym9995@163.com (Y.H.); ancher0928@163.com (C.A.); chengyan1220@hotmail.com (Y.C.); yanmaokai123@163.com (M.Y.); chaigaifeng@163.com (G.C.); xpniu0613@126.com (X.N.); caihanyang123@163.com (H.C.); 2 Horticulture Research Institute, Guangxi Academy of Agricultural Sciences, Nanning Investigation Station of South Subtropical Fruit Trees, Ministry of Agriculture, Nanning 530007, China; dengbiao3333@163.com (B.D.); lym@gxaas.net (Y.L.); 3 College of Plant Science & Technology, College of Life Science and Technology, Huazhong Agricultural University, Wuhan 430070, China; junzhangl@outlook.com; 4 College of Plant Protection, Fujian Agriculture and Forestry University, Fuzhou 350002, China; 5 Institute of Horticultural Biotechnology, College of Life Sciences, Fujian Agriculture and Forestry University, Fuzhou 350002, China; zhengping13@mails.ucas.ac.cn

**Keywords:** self-incompatibility, *Passiflora edulis* f. *flavicarpa*, transcriptome, time-course analysis

## Abstract

Self-incompatibility (SI) is an important plant mechanism that prevents inbreeding depression by recognizing and rejecting self-pollen, thereby promoting outcrossing. However, SI can also act as a barrier in breeding programs, presenting significant challenges to breeders. Passion fruit (*Passiflora edulis*), a tropical fruit species of substantial economic importance, also serves as a valuable system for investigating SI mechanisms within the Passifloraceae. Nevertheless, the molecular basis of SI in passion fruit has not yet been elucidated. In this study, we investigated the SI system in yellow passion fruit (*P. edulis* f. *flavicarpa*) and employed transcriptomic analysis to examine the time-course transcriptional responses following different pollination treatments. Transcriptomic analysis revealed distinct gene expression dynamics under different pollination treatments: self-pollinated samples exhibited stronger and earlier transcriptional changes, whereas the number of differentially expressed genes (DEGs) in cross-pollinated samples was relatively lower. Numerous pathways previously associated with sporophytic self-incompatibility (SSI) were enriched in the stigma samples after self-pollination. Reactive oxygen species (ROS) are crucial signaling molecules involved in pollen germination and pollen tube growth during SI responses. Our results showed that ROS-related pathways were enriched in stigma tissues after self-pollination. In addition, oxidative stress-related responses were detected in the style shortly after self-pollination, suggesting that plastid-associated or general oxidative stress processes may also be involved, although the precise source of ROS requires further validation. *FERONIA*, *ROP9*, and *ARC1* are key genes related to the SI system in *Brassica*. In the passion fruit SI response, the expression levels of these genes increased in the style, indicating a spatial expression pattern different from that reported in classical *Brassicaceae* SSI systems. Together with cytological observations showing that self-pollen rejection occurs at the stigma surface, our results suggest that yellow passion fruit may employ an SSI-like regulatory framework while exhibiting a lineage-specific spatial deployment of SI-related regulators. Overall, this study provides new transcriptomic insights into the SI mechanism of yellow passion fruit, establishes a molecular framework for understanding SI in *P. edulis* f. *flavicarpa*, and offers novel insights into the diversity of plant SI systems.

## 1. Introduction

Passion fruit (*Passiflora edulis*), a member of the Passifloraceae family and the Passiflora genus, is a perennial woody vine extensively cultivated in tropical and subtropical regions. It is widely appreciated for its distinctive aroma, abundant juice, and high nutritional value, making it a commercially important crop with a substantial market value [1,2,3]. Edible passion fruit is generally classified into purple passion fruit (*Passiflora edulis Sims*) and yellow passion fruit (*Passiflora edulis* f. *flavicarpa*). Purple passion fruit is native to America and is mainly adapted to higher elevations, whereas yellow passion fruit, which is suitable for lower elevations, is believed to be either a mutation of the purple type or a natural hybrid between purple passion fruit and another related Passiflora species [1]. Yellow passion fruit exhibits superior agronomic traits, such as a stronger aroma, greater disease resistance, and a larger fruit size, making it a preferred parent in breeding programs. However, most yellow passion fruit germplasm exhibits self-incompatibility, which poses a major challenge to breeding efforts.

Self-incompatibility mechanisms in flowering plants prevent self-fertilization by specifically distinguishing between self- and non-self-pollen [4]. In most angiosperms, SI systems are controlled by a highly variable S-locus, which contains at least two closely linked genes responsible for mediating self/non-self recognition [5]. In some species of the Poaceae family, however, SI is controlled by more than one locus. SI systems are generally classified as sporophytic or gametophytic according to the genetic control of the pollen SI phenotype. Previous studies have extensively explored these SI systems at the molecular level. Three primary types have been identified: (1) gametophytic SI (GSI) based on the SLF (SFB)/SRNase system in the *Solanaceae*, *Rosaceae*, and *Plantaginaceae* [6,7,8,9,10], (2) gametophytic SI based on the PrpS/PrsS system in the *Papaveraceae* [11], and (3) sporophytic SI (SSI) based on the SP11 (SCR)/SRK system in the *Brassicaceae* [12].

In the *Brassicaceae*, SSI is controlled by the S-locus, which contains two highly polymorphic and tightly linked genes that enable the stigma to recognize and reject self-pollen [13]. One of these genes encodes a serine/threonine receptor kinase, S-locus receptor kinase (SRK), which is predominantly expressed in the stigma epidermis and functions as the female S-determinant [14]. The second gene encodes a cysteine-rich protein, S-locus cysteine-rich protein/S-locus protein 11 (SCR/SP11), which serves as the pollen ligand of SRK [15]. SCR/SP11 determines SI specificity in pollen, whereas a third gene, S-locus glycoprotein (SLG), may enhance SI expression [12]. SCR and SRK proteins exhibit high sequence divergence among different haplotypes, particularly in the extracellular S domain. When SCR/SP11 and SRK from the same S-haplotype interact specifically, the SI signaling pathway is activated in stigma papilla cells [16,17,18,19]. In addition to S-locus genes, several downstream regulators have been identified in the *Brassicaceae* SI signaling pathway. M-locus protein kinase (MLPK) plays a key role by interacting with SRK to transmit SI signals [20]. SRK also phosphorylates the ARM-repeat and U-box protein ARC1, which then ubiquitinates Exo70A1, a component involved in vesicle trafficking [21,22]. The kinase domain of SRK interacts with THIOREDOXIN H-LIKE proteins (THL1 and THL2), which inhibit SRK activity in the absence of the SP11/SCR ligand [23]. ARC1 is an E3 ubiquitin ligase and one of the best-characterized downstream effectors of SRK. The absence or suppression of ARC1 can lead to the breakdown of SSI and result in self-compatibility (SC). Upon activation, ARC1 targets compatibility factors for ubiquitination and degradation [24]. Exo70A1, a subunit of the exocyst complex necessary for pollen hydration, acts as a compatibility factor, and its overexpression in Brassica can compromise the SSI response [25].

Recent studies have identified additional ARC1 targets in *Brassica*, including glyoxalase 1 (GLO1) and phospholipase D alpha 1 (PLDα1), both of which function as stigma compatibility factors [26,27]. GLO1 is crucial for detoxifying methylglyoxal, whereas PLDα1, which is enriched in stigmas, is essential for compatible pollen responses and may produce phosphatidic acid (PA) to facilitate exocytosis from papilla cells. Overexpression of GLO1 and PLDα1 in self-incompatible Brassica napus leads to the breakdown of the SI response [26,27]. Recent studies have also revealed several key downstream processes involved in SI responses [13,28,29]. Reactive oxygen species (ROS), which play important roles in pollen tube growth, are highly accumulated during the SI response in Brassica. This process depends on signaling mediated by the FERONIA (FER) receptor kinase and Rac/Rop GTPases [30,31,32]. Furthermore, phytohormones are closely associated with SI stability, as shown in *Arabidopsis* and *Brassica* species [13,28,29]. For instance, in Chinese cabbage, ethylene negatively regulates SI by inducing programmed cell death (PCD) in stigmatic papilla cells [29]. In addition, the growth regulator auxin interacts with SI signaling through Auxin Response Factor 3 (ARF3) [28].

For decades, the *Brassicaceae* SI system has served as a model for studying SI mechanisms in passion fruit. However, genetic analyses and microscopic evidence indicate that the SI system in passion fruit differs from that of Brassica species [33,34,35]. Recent molecular and omics studies have advanced our understanding of the SI in *Passiflora edulis,* including the cloning of an SLG-like fragment and the identification of SRK and SLG homologs in the wild diploid species *Passiflora* organensis [36,37]. Despite these advances, the molecular basis of SI in passion fruit remains largely unknown. To investigate the SI mechanism and identify candidate genes associated with SSI-like responses in yellow passion fruit, we conducted self-pollination (‘Baxi’ × ‘Baxi’, SP), cross-pollination (‘Baxi’ × ‘TN-1’, CP), and cut-stigma self-pollination (‘Baxi’ × ‘Baxi’, CSP) experiments, followed by RNA-seq analysis. Our results revealed distinct transcriptional profiles among different pollination treatments. Interestingly, several genes known to participate in the SI response in Brassica were preferentially activated in the styles of self-pollinated passion fruit. These findings provide transcriptomic evidence that yellow passion fruit may employ an SSI-like regulatory framework with a lineage-specific spatial deployment of SI-related genes, thereby offering new insights into the molecular basis of SI in *P. edulis* f. *flavicarpa*.

## 2. Result

### 2.1. The Pollen Tube Growth Observation

**Figure 1:**
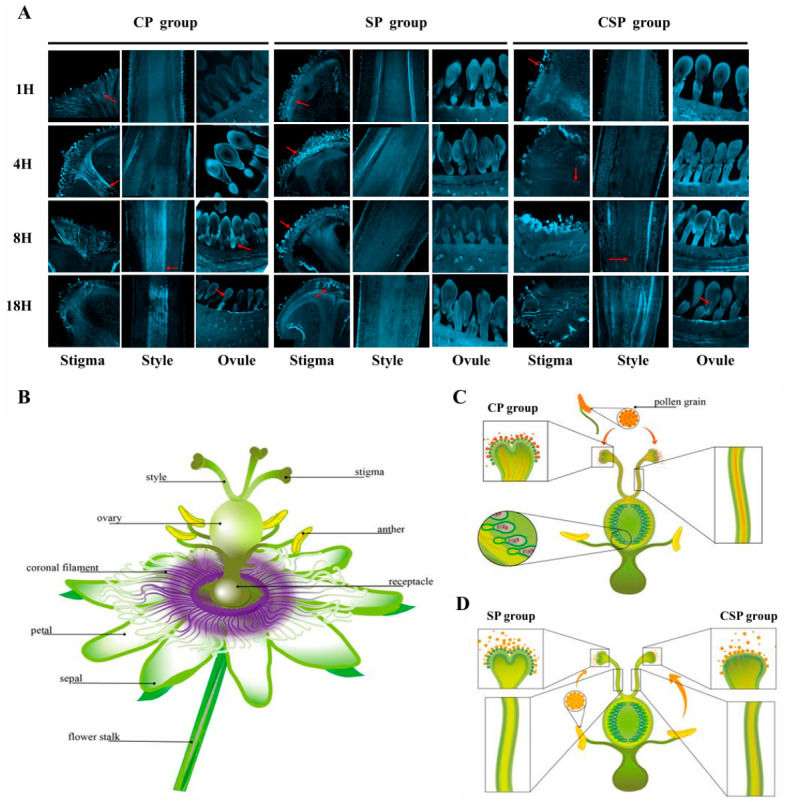
Micrographs of pollen–stigma interactions and pollen tube behavior in yellow passion fruit. (**A**) Confocal microscope observation results of compatible pollen and pollen tube behavior at 1, 4, 8 and 18 h after pollination (HAP). The red arrow indicates pollen and pollen tubes (**B**) Schematic diagram of yellow passion fruit flower structure. (**C**) Pollen–stigma interactions during cross-pollination. In the CP group, compatible pollen germinated and reached the ovule; (**D**) Pollen–stigma interactions under two different pollination treatments. In the SP group, incompatible pollen failed to germinate and did not reach ovule. In the CSP group, only a few incompatible pollen germinated and reached the ovule. CP, cross pollination; SP, self-pollination; CSP, cut-papilla self-pollination.

In this study, field-grown plants of the cultivar ‘Baxi’ were used to investigate self-incompatibility (SI) in yellow passion fruit. We conducted self-pollination (‘Baxi’ × ‘Baxi’, SP), cross-pollination (‘Baxi’ stigmas pollinated with compatible ‘TN-1’ pollen, CP), and cut-stigma self-pollination (‘Baxi’ × ‘Baxi’, CSP) experiments. Pollen behavior following different pollination treatments was observed using confocal microscopy. The results showed that pollen germination occurred in both the CP and CSP groups (Figure 1). In the CP group, pollen exhibited a high germination rate, with pollen tubes penetrating the stigma within 1 h and reaching the top of the style within 4 h (Figure 1A,C). At 8 h, the pollen tubes had passed through the style and entered the ovule within 18 h (Figure 1A). In contrast, under CSP treatment, only a small number of pollen tubes penetrated the stigma and extended into the style (Figure 1A,D). Conversely, in the SP group, pollen tubes were inhibited on the stigma surface and failed to germinate (Figure 1A,D). These results show that self-pollen could partially germinate after the removal of the stigma papilla cells by the cut-stigma treatment, indicating that the SI response in yellow passion fruit is initiated mainly at the stigma surface. This pattern is similar to the sporophytic self-incompatibility (SSI) characteristics observed in the *Brassicaceae* and is consistent with previous studies on yellow passion fruit [30,36]. Therefore, our cytological observations suggest that yellow passion fruit may exhibit an SSI-like pollen rejection pattern, although its underlying molecular mechanism requires further investigation.

### 2.2. Different Pollination Treatments Induce Distinct Transcriptional Profiles

**Figure 2:**
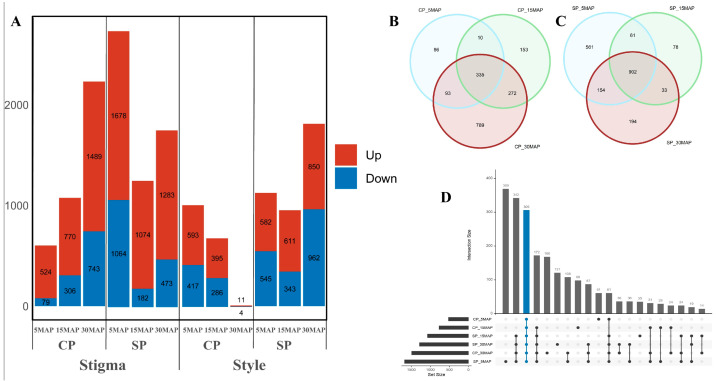
Comparison of gene expression levels of stigma and style under CP and SP of yellow passion fruit. (**A**) DEGs identified in CP and SP samples in stigma and style. (**B**,**C**) Venn diagram of up-regulated DEGs in CP-stigma and SP-stigma groups; (**D**) Graph of up-regulated DEGs in stigma groups. The blue dots represent all time points. The black dot represents specific time point. CP, cross pollination; SP, self-pollination.

To explore the molecular mechanisms underlying SI in yellow passion fruit, we collected stigma and style tissues from three experimental groups: unpollinated controls, the CP group, and the SP group. Samples from the CP and SP groups were harvested at 5, 15, and 30 MAP (minutes after pollination) and subjected to transcriptomic analysis. Differential expression analysis was conducted using edgeR, with genes exhibiting an absolute log2 fold change ≥ 1 and a false discovery rate (FDR) ≤ 0.05 defined as differentially expressed genes (DEGs). Unpollinated samples (0 MAP) were used as controls for comparisons at each time point. The analysis revealed distinct transcriptional dynamics between the CP and SP groups (Figure 2A). In the stigma, the CP group showed a progressive increase in DEG numbers over time, with 603, 1076, and 2232 DEGs identified at 5, 15, and 30 MAP, respectively. In contrast, the SP group exhibited an abrupt early transcriptional shift, with 2742, 1256, and 1756 DEGs detected at the same time points (Figure 2A). A similar divergence was observed in style tissues. The CP-style group showed a rapid decrease in DEG numbers, from 1010 at 5 MAP to 681 at 15 MAP and only 15 at 30 MAP (Figure 2A). Conversely, the SP-style group showed an early surge, with 1127 DEGs at 5 MAP, and reached a peak of 1812 DEGs at 30 MAP, showing a dynamic pattern similar to that observed in the CP-stigma group (Figure 2A). In stigma samples, a total of 335 up-regulated DEGs were identified across all time points in the CP group, whereas 902 were identified in the SP group, with 306 DEGs commonly up-regulated in both groups (Figure 2B–D). In style tissues, 207 up-regulated DEGs were consistently detected across all time points in the SP group, whereas 188 were detected in the CP group, excluding the 30 MAP samples because of the very low number of DEGs. Across all pollination treatments, the number of up-regulated DEGs markedly exceeded that of down-regulated DEGs (Figure 2A). These expression patterns suggest that both the stigma and style respond rapidly and sensitively to pollination stimuli. Moreover, the divergence in transcriptional profiles implies that different types of pollen may activate distinct signaling pathways.

### 2.3. Enrichment Analysis Reveals Key Biological Processes Associated with SI in Yellow Passion Fruit

**Figure 3:**
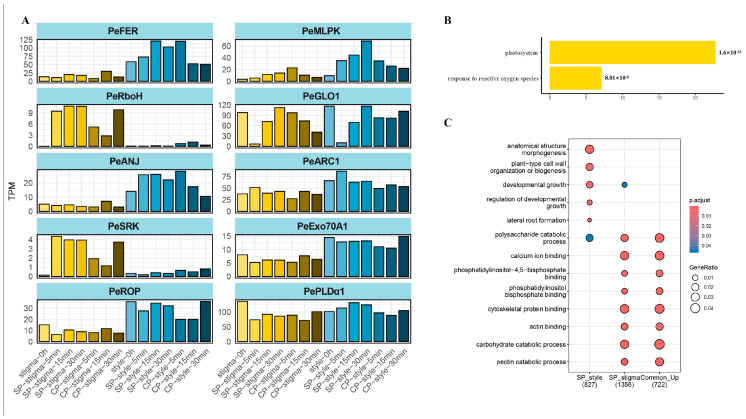
Expression profiles and enrichment results indicating that yellow passion fruit may use different SI systems. (**A**) The expression profile of the homolog of key genes from the *Brassicaceae* SI system. (**B**) The enrichment result of 0 Vs. 5 MAP style in the SP group. (**C**) The enrichment result of down-regulated DEGs in 0 Vs. 30 MAP style (SP_style), up-regulated DEGs in 0 Vs. 5 MAP stigma (SP_stigma), and up-regulated DEGs in all SP-stigma. The size of the circle represents the genes ratio in different biological pathways. Different colors indicate adjusted *p*-value.

Several physiological responses have been reported following the deposition of self-pollen in self-incompatible species, including elevated cytosolic calcium levels [38], protein phosphorylation [13], depolymerization of actin and microtubules [39,40,41], and increased ROS accumulation in stigmatic tissues [30]. To gain deeper insights into the biological processes underlying the SI response, we performed functional enrichment analysis of DEGs. Based on the above transcriptional results, we focused on two key comparisons that showed notable changes: the 5 MAP SP-stigma group and the 30 MAP SP-style group. In the 5 MAP SP-stigma group, up-regulated DEGs were significantly enriched in gene ontology (GO) terms related to “cytoskeleton”, “calcium ion binding”, “enzyme regulator activity”, “pectin catabolic process”, “pollen tube growth”, “pollination”, and “proton transmembrane transporter activity” (Figure 3A). These results indicate that SI-related pathways are activated at a very early stage following self-pollination. In contrast, down-regulated DEGs in this group were enriched in terms associated with “ribosome biogenesis”, “DNA replication”, and “ncRNA processing” (Table S1), suggesting that growth- and proliferation-related activities may be suppressed during the initial SI response.

In the 30 MAP SP-style group, up-regulated DEGs were predominantly enriched in hormone-activated signaling pathways, including “response to auxin”, “signaling receptor activity”, and “response to ethylene” (Table S1). These pathways have previously been implicated in SI responses in other species, implying that hormone-related regulation may also participate in the SI response of passion fruit [28,29]. Conversely, down-regulated DEGs were specifically enriched in morphogenesis-related terms, such as “anatomical structure morphogenesis”, “lateral root formation”, and “plant-type cell wall organization” (Table S1). Given the well-documented similarities between tip growth in pollen tubes and lateral roots [42,43], this enrichment pattern suggests that cellular processes associated with tip growth may be repressed during the SI response, thereby contributing to pollen tube inhibition. Further evidence was obtained from the enrichment results of the 30 MAP CSP-style group, in which no root-related pathways were enriched among down-regulated DEGs. The expression profile showed that the down-regulated genes in the 30 MAP SP-style group were predominantly expressed in unpollinated styles (0 MAP) and in compatible pollination samples (30 MAP CP-style). This gene set included six expansins, three acyl-CoA synthetases, several kinases, and one MYB transcription factor (Table S2), pointing to a potential regulatory network associated with pollen tube elongation under SI conditions.

### 2.4. Comparative Analysis of Stigma Transcriptomes Reveals Both Shared and Pollination-Specific Responses

Among the up-regulated DEGs in CP and SP stigma samples across all time points, 306 genes were commonly induced. These shared DEGs were primarily enriched in GO terms related to cell wall modification and signaling, including “enzyme regulator activity”, “pectin catabolic process”, “cell wall modification”, “protein autophosphorylation”, “oxidoreductase activity”, and “cytoskeleton” (Table S1). Many of these processes are known to be involved in root hair and pollen tube growth, suggesting that common cellular machinery is activated after pollination regardless of pollen compatibility. In contrast, the 902 DEGs specifically and consistently up-regulated in SP-stigma samples were enriched in terms such as “pectin catabolic process”, “pollination”, “cytoskeleton”, “chloroplast”, “pollen tube growth”, and “protein tyrosine kinase activity” (Figure 3C). Notably, most of these genes exhibited high expression levels in all SP-stigma samples but peaked only at 30 MAP in the CP-stigma group, indicating distinct temporal dynamics between compatible and incompatible interactions. Among the SP-stigma-specific DEGs, we identified a candidate SRK-like gene, PassEdu10265. However, phylogenetic analysis revealed that this gene is evolutionarily distant from canonical *Brassicaceae* SRK homologs (Figure 3A and Figure S1), suggesting that although similar signaling components may be involved, the molecular basis of SI in yellow passion fruit may differ from the well-characterized *Brassicaceae* system. Additionally, in the 15 MAP CP-stigma group, terms related to “ribosome”, “water channel activity”, and “cell wall organization” were significantly enriched. In the SP-stigma group at the same time point, down-regulated DEGs were enriched in “starch metabolic process” and “polysaccharide metabolic process”. At 30 MAP, down-regulated DEGs in the CP-stigma group were associated with “rRNA metabolic process”, “ncRNA processing”, and “DNA replication”, whereas in the SP-stigma group, terms such as “methylation”, “lateral root formation”, and “cellular carbohydrate metabolic process” were overrepresented (Table S1).

Taken together, these enrichment results demonstrate that both self- and cross-pollen trigger transcriptional reprogramming in the stigma and style, activating both shared and distinct pathways. Early activation of SI-related pathways occurs in the stigma within 5 min after self-pollination, accompanied by the suppression of growth-related processes. Hormone signaling involving auxin and ethylene becomes prominent in the style during later stages of the SI response. Concurrently, several down-regulated DEGs in the style were associated with cell wall organization, cell wall modification, and growth-related processes, suggesting that tip growth-related cellular processes may be affected during the SI response. Comparative analysis further revealed shared pollination responses related to cell wall modification and signaling, alongside SP-specific pathways involving cytoskeletal dynamics and oxidative stress. In addition, the identification of a candidate SRK-like gene phylogenetically distinct from *Brassicaceae* homologs suggests a divergence in the molecular mechanism underlying SI in passion fruit. Collectively, these enrichment patterns highlight the dynamic and tissue-specific reprogramming triggered by self-pollination and provide a transcriptomic framework for understanding the SI mechanism in yellow passion fruit.

### 2.5. ROS May Act as a Key Factor in Passion Fruit SI

**Figure 4:**
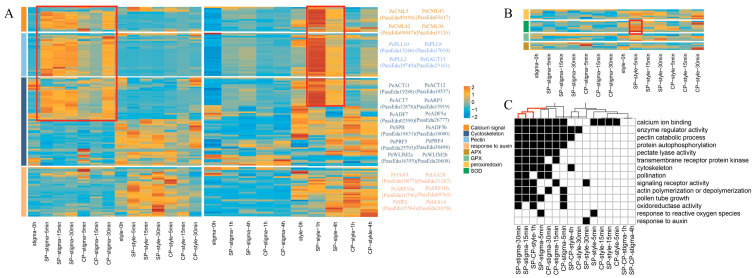
(**A**,**B**) The expression profile of chloroplast ROS production and SI-related pathways, the red box indicates that calcium signaling, pectin and parts of cytoskeleton genes were expressed specifically in SP style, and this trend may be maintained for hours. (**C**) Enrichment of GO terms for DEGs in each comparison groups; the red line indicates that 15, 30 MAP and 1HAP SP style comparison groups.

GO enrichment analysis of style samples revealed striking differences between pollination treatments. At 5 MAP in the CP-style group, up-regulated DEGs were enriched in pathways related to “calcium signaling”, “cellular carbohydrate metabolic process”, “phosphatase activity”, “response to abiotic stimulus”, and “transporter activity” (Figure 4B, Table S1). In contrast, in the 5 MAP SP-style group, up-regulated DEGs were primarily associated with responses to abiotic stimuli, including “reactive oxygen species”, “osmotic stress”, as well as “thylakoid” and “photosystem” processes (Figure 3B, Table S1). Similarly, at 30 MAP, up-regulated DEGs in the SP-style group were mainly enriched in “response to oxygen-containing compound”, “auxin-activated signaling pathway”, and “regulation of signal transduction” (Table S1). The enrichment of ROS-related and oxidative stress-related terms in post-pollination style tissues prompted us to further investigate the molecular basis of this response.

To investigate whether conserved regulators, including FERONIA, MLPK, and ARC1 [20,22,24,26,27,30], participate in the passion fruit SI response, we characterized their homologs and examined their expression profiles (Figure 3A). Several genes maintained high expression levels in the style, including *PeFER*, *PeMLPK*, *PeGLO1*, *PeANJ*, and *PeROP* (Figure 3A). MLPK, which transduces SI signaling specifically in the stigma of *Brassica* [20,44], showed gradually increasing expression over time in the SP group. FERONIA, ROP, and ANJEA have been reported to activate downstream signaling for ROS production [30,45]. In this study, these genes maintained high abundance specifically in the SP-style samples, showing a spatial expression pattern different from that reported in the classical *Brassicaceae* SSI system. The contrasting downstream responses of the CP and SP styles, along with the expression dynamics of these key regulators, suggest that ROS-related signaling may be specifically activated in SP-style tissues (Figure 3A–C).

Previous studies have demonstrated that ROS play a key role in both sporophytic (SSI) and gametophytic (GSI) self-incompatibility systems [30,46]. Our results showed that ROS-related genes were activated following the SI response in passion fruit. At the earliest time point (5 MAP), the GO term “response to hydrogen peroxide” was highly enriched in both the CP- and SP-stigma samples (Table S1). This term included four small heat shock protein genes in the CP-stigma samples and nine in the SP-stigma samples, which are known to be expressed in response to oxidative stress [47]. We also identified one *Rboh* gene, *PassEdu04091*, that maintained consistently high expression from 0 to 30 MAP specifically in the SP-stigma samples (Figure S2). However, *PassEdu00364* and *PassEdu18055* were specifically expressed in the CP-stigma samples (Figure S2), suggesting differential regulation of ROS-producing enzymes depending on pollen compatibility. Additional evidence for oxidative stress was found specifically in the SP-style tissues (Table S1). GO terms such as “photosystem”, “response to reactive oxygen species”, “thylakoid”, “response to oxygen-containing compound”, and “response to hydrogen peroxide” were significantly enriched in the SP-style group (Figure 3B, Table S1). The enrichment of photosynthesis- and thylakoid-related terms in SP-style samples suggests that plastid-associated oxidative processes may be involved in the SI response. However, because style tissues are not typically considered highly photosynthetic, these results should not be interpreted as direct evidence for chloroplast-derived ROS without further experimental validation.

Therefore, we further characterized key enzymes potentially involved in plastid-associated ROS metabolism in yellow passion fruit. Chloroplast-associated superoxide dismutases (SODs), including iron-SODs and copper/zinc-SODs, catalyze the dismutation of superoxide radicals into hydrogen peroxide. We identified nine candidate SOD genes, three of which—*PassEdu06577*, *PassEdu22761*, and *PassEdu15664*, were up-regulated in SP-style samples (Figure 4B; Table S2). Notably, *PassEdu06577*, the homolog of *Arabidopsis AtCSD3*, was up-regulated in both comparison groups. The expression profiles of SOD genes showed that most of them (six of nine) were highly expressed in the 5 MAP SP-style samples. Hydrogen peroxide can be detoxified by ascorbate peroxidases (APXs), glutathione peroxidase-like enzymes (GPXLs), and peroxiredoxins. Our results showed that four of six candidate GPXL genes were highly expressed in SP-style tissues from 5 to 30 MAP, with PassEdu08234 and PassEdu22570 reaching peak expression at 5 MAP and 30 MAP, respectively (Figure 4B; Table S2). The coordinated up-regulation of SOD and GPXL genes specifically in the SP-style samples supports the involvement of oxidative stress-related processes in the SI response.

In conclusion, our results suggest that the ROS may maintain high levels in both the stigma and style tissues during the SI response, but the production pathway varies depending on the tissue type. In the stigma, Rboh genes appear to contribute to ROS production, while in the style, the enrichment of chloroplast-related terms and the expression patterns of *SOD* and *GPXL* genes point toward thylakoid-derived ROS. The homologs of key regulators involved in ROS signaling—*PeFER*, *PeROP*, and *PeANJ*—exhibited high expression levels in style tissues at early stages following self-pollination, coinciding with the enrichment of oxidative stress-related GO terms and the up-regulation of *SOD* genes. These findings support the hypothesis that ROS signaling, which plays an important role in regulating pollen germination, is activated specifically in the SP-style during the self-incompatibility response in passion fruit. Further research is needed to understand the impact of these potentially high ROS levels, given their dual role in pollen tube growth.

### 2.6. Investigation of Other SI-Related Biological Processes in Yellow Passion Fruit

Upon self-pollen deposition, plants activate various biological pathways to prevent self-fertilization. Our transcriptomic data revealed dynamic changes in several key processes beyond ROS signaling, prompting us to investigate their potential roles in passion fruit SI. Actin cytoskeleton depolymerization is considered a major factor in the SI response across multiple species [39,41,48]. The dramatic depolymerization and rearrangement of the cytoskeleton in pollen tubes can trigger programmed cell death (PCD) during SI. To determine whether this process is conserved in passion fruit, we characterized genes related to actin depolymerization, including actin, ADF (actin depolymerizing factor), FIM (fimbrin), LIM, and profilin. Among 17 actin genes, five genes (*PassEdu19288*, *PassEdu19537*, *PassEdu28906*, *PassEdu00958*, and *PassEdu02294*) were highly expressed in stigma tissue in both the SP and CP groups (Figure 4, Table S2). These genes were mainly activated 30 MAP in the CP group but maintained consistent expression across all time points in the SP group. Four of 17 *ADFs* (*PassEdu10234*, *PassEdu00010*, *PassEdu02599* and *PassEdu12362*) showed increased expression in the CP-stigma samples, but remained consistently activated in the SP-stigma samples. Two additional *ADFs* (*PassEdu09668* and *PassEdu19571*) peaked at 5 MAP in the CP-stigma, with no *ADF* genes showing specific expression in the CP-style tissues (Figure 4, Table S2). This expression pattern was also observed in *Fimbrin*, *LIM*, and *Profilin* (Figure 4, Table S2). This result demonstrated that cytoskeleton dynamics are triggered by the SI response in stigma tissues and related genes generally peaked at 30 MAP. However, distinct temporal patterns emerged between treatments: SP samples maintained high gene abundance throughout the experiment, while CP samples showed progressively increasing expression.

**Figure 5:**
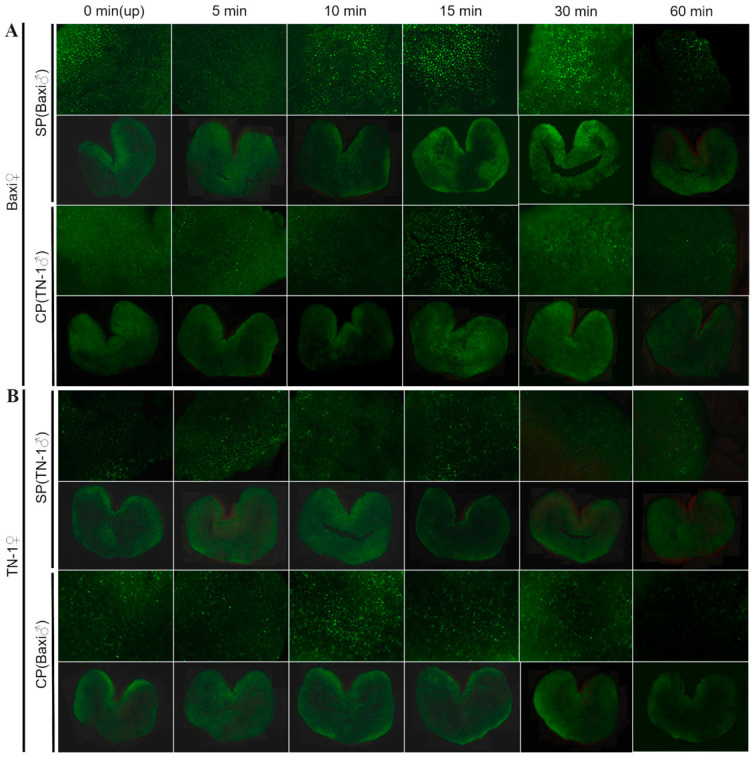
Analysis of ROS levels in the stigma of SP and CP in different varieties of passion fruit. (**A**) Analysis of ROS levels in the stigma of SP and CP in the ‘BX’ passion fruit. (**B**) Analysis of ROS levels in the stigma of SP and CP in the ‘TN-1’ passion fruit. up: unpollinated; SP: self-pollination; CP: cross-pollination.

We also observed that Ca^2+^-responsive genes *PeCML3*, *PeCML42*, *PeCML16*, *PeCML25* showed increased expression patterns in both the SP and CP stigma tissues, although their expression levels were different (Figure 4). Notably, two *CML* (calmodulin-like) genes (*PeCML15* and *PeCML37*) and two *CPK* (calcium-dependent protein kinase) genes (*PeCPK20* and *PeCPK17*) showed specific expression in the SP-style tissues. The *Arabidopsis* homologs of *PeCML15*, *PeCPK20* and *PeCPK17* have been reported to be specifically expressed in the anther and pollen grains and are implicated in regulating pollen tube growth [49,50,51]. In contrast, the homolog of *PeCML37* functions as a regulator of Ca^2+^ signaling during plant–pathogen interactions, mediating jasmonate and abscisic acid signaling [52]. Genes involved in the metabolic processes of the cell wall of stigma papilla cells after pollination, such as *PePPME1* (*PassEdu02744*), *PePMEI5* (*PassEdu10522*), and *PeCOG3* (*PassEdu25926*), which belong to the PME, PMEI, COG, PLL, and GAUT families, showed sustained high expression in the SP-stigma samples but were highly expressed only at 30 MAP in the CP-stigma samples (Figure 5A; Table S2). This distinct expression pattern suggests that the molecular control of pollen tube germination may differ between compatible and incompatible pollination, or alternatively, that SI determinants may reject self-pollen through a dosage-dependent mechanism.

The expression patterns of auxin-related genes were also identified (Figure 5, Table S2). The homologs of *PeAUX2-11* (*PassEdu20159*), *PeARF16* (*PassEdu16796*), *PeARF19* (*PassEdu30656*), SAURs and IAAs were generally expressed in style tissues and preferentially expressed in the SP samples across all time points, displaying similar expression levels in the early stages (5 and 15 MAP) of the CP samples (Table S2). The expression profile of the *ARF3* homolog (*PassEdu06037*) maintained high expression levels in style samples from both the CP and SP groups, with no significant difference observed between the two groups (Table S2). The expression profiles of these auxin-related genes suggest that auxin may play a crucial role in the passion fruit SI response, similar to its role in the *Brassicaceae*, indicating the potential conservation of the molecular mechanism. Root hairs and pollen tubes employ conserved pathways to direct growth, including cytoskeleton organization, membrane trafficking, calcium signaling pathways, phosphoinositide, ROPs and ROS [41,53,54,55,56,57,58]. Among the DEGs annotated as root morphogenesis-related, three homologs of *LACS2* (*PassEdu31783*, *PassEdu04927*, and PassEdu26931), *PeMRI* (*PassEdu00984*), *PeMKK6* (*PassEdu25792*) were identified. The transcriptomic dynamics and high expression of genes in certain pathways further indicated the potential role of style tissues in the SI mechanism.

To gain deeper insight into style-specific responses, we further sampled and sequenced stigma and style tissues at 1 and 4 HAP. The calcium signaling pathways, phosphoinositide, cytoskeleton and other pathways were activated in the 1 HAP SP-style (Figure 4C). The enriched pathways and expression profiles were highly similar to those of the 5 MAP SP-stigma (Figure 4C). For example, the GO term “calcium ion binding” included 47 DEGs in the 5 MAP SP-stigma samples and 41 DEGs in 1 HAP SP-stigma samples, and 27 DEGs overlapped. A similar result was observed in “cytoskeleton” (39 DEGs in 5 MAP, 31 DEGs in 1 HAP, 26 DEGs were shared), “phosphatidylinositol binding” (16 DEGs in 5 MAP, 14 DEGs in 1 HAP, 13 DEGs were shared), “cell tip growth” (9 DEGs in 5 MAP, 7 DEGs in 1 HAP, 6 DEGs were shared) and other terms (Figure 4C). These results suggest that the transcriptomic dynamics in the early stage of the SP-stigma and later stage of the SP-style are remarkably similar, further supporting our hypothesis that style tissues actively participate in the SI response through conserved signaling pathways.

### 2.7. Transcription Factors May Play a Crucial Role in SI Response in Passion Fruit

To investigate the potential role of TFs during the SI response, the exclusively up-regulated DEGs in the 5 MAP SP group were extracted and characterized using iTAK, a powerful program for TF identification [59]. A total of 70 TFs were identified, including 10 genes from each of the AP2/ERF and MYB families, five genes from each of the MADS-box, bZIP, HD-ZIP, and WRKY families, four genes from the C2H2 family, three genes from the NAC and LOB families, two genes from each of the bHLH, TUB, NF-YB, GARP-G2-like, and GRAS families, and single representatives from the HB-BELL, FAR1, HB-PHD, B3, C3H, C2C2-GATA, and C2C2-Dof families (Table S3). Further analysis showed that two AP2/ERF family members (*PassEdu02629* and *PassEdu25907*) were specifically expressed in style tissues, suggesting potential roles in style-mediated SI responses. Additionally, a bZIP family member (*PassEdu30917*), homologous to *AtPCMP-H80*, was highly expressed at 5 MAP in the SP-stigma samples, suggesting a potential role in the early stages of the SI response. Our findings indicate that TFs are activated after pollination, particularly in the SP group. The expression profiles of many of these transcription factors (TFs) are similar to those of other SI-related genes, suggesting their potential involvement in mediating downstream signaling events during SI.

### 2.8. Exploring the Role of ROS in the SI Response of Passion Fruit

To investigate whether ROS are involved in the SI response of passion fruit, our study utilized the general ROS probe H_2_DCFDA to observe the stigma of passion fruit at different time points after SP and CP through staining. The SI variety ‘Baxi’ was used as the maternal parent to examine ROS levels on the stigma surface under conditions of SP and CP (using ‘TN-1’ as the paternal parent). These results showed that in the SI variety ‘Baxi’, the fluorescence intensity on the stigma surface at 5 min and 10 min after SP was essentially consistent with that of the unpollinated control, with no significant changes. At 15 min after pollination, the fluorescence intensity on the stigma surface increased, reaching its peak at 30 min after pollination, and then decreased to the level of the unpollinated control at 60 min after pollination (Figure 5A). However, following CP of ‘Baxi’ (using ‘TN-1’as the paternal parent), no significant change in fluorescence intensity was observed on the stigma surface, and no increase in fluorescence intensity was detected (Figure 5A). These results indicate that under SP, the ROS level on the stigma surface of the SI variety ‘Baxi’ is higher than that under CP, suggesting that ROS participate in the SI response of passion fruit and remain at a relatively high level during this response. Combined with the previously obtained expression profiles of SI-related genes in the stigma of ‘Baxi’, these results speculate that the ROS response on the stigma surface mediates the SI process in passion fruit. In addition, our study also used ‘TN-1’ passion fruit as the maternal parent to observe ROS levels on the stigma surface under SP and CP (using ‘Baxi’ as the paternal parent). The results showed that there was no significant difference in ROS levels on the stigma surface of ‘TN-1’ between SP and CP. Moreover, the ROS levels on the ‘TN-1’ stigma after SP and CP remained essentially unchanged compared to those on the unpollinated stigma (Figure 5B). These findings indicate that ROS levels on the stigma surface of ‘TN-1’ remained unaffected by SP or CP.

## 3. Discussion

Self-incompatibility (SI) acts as a fundamental reproductive barrier that prevents inbreeding depression in flowering plants, while simultaneously posing a major obstacle for crop improvement. Elucidating the molecular basis of SI is therefore of both theoretical and practical importance. Although previous studies have proposed both sporophytic and gametophytic control models in passion fruit, robust molecular evidence remains limited [36,60]. In this study, we combined cytological observations with time-resolved transcriptomic analyses to provide a comprehensive view of the SI responses in yellow passion fruit.

Microscopic observations clearly demonstrated that self-pollen failed to penetrate the stigma surface, whereas compatible pollen germinated and successfully reached the ovule, supporting the stigma as the primary site of SI determination. The partial recovery of pollen germination in the cut-stigma treatment further suggests that, in addition to structural barriers, local physiological factors such as oxidative status or hydration conditions may also contribute to pollen rejection. These results indicate that the papillae are one of the key sites for controlling the sporophyte. In the typical *Brassicaceae* SSIs, rejection reactions occur on the surface of the stigma, and mastoid cells participate in recognizing and preventing the hydration of incompatible pollen. After removing the papillae, incompatible pollen gained the ability to hydrate and grew pollen tubes [30]. Together, these findings shoe similarities to the *Brassicaceae* SSI system while also indicating potential deviations from the classical SSI system. However, during the process of removing the papillae, the natural barrier that recognizes hydration on the plant stigma was disrupted.

Transcriptomic analyses revealed striking differences between compatible and incompatible pollination responses. Self-pollination triggered a rapid and extensive transcriptional reprogramming in the stigma, whereas cross-pollination induced a more gradual response. This early and strong activation pattern suggests that self-pollen may be recognized as a stress-like signal, rapidly initiating defense-associated pathways. Consistent with this interpretation, genes related to calcium signaling, cytoskeleton dynamics, and reactive oxygen species (ROS) were significantly enriched in the early stages of the SI response, indicating that these conserved signaling modules may play central roles in pollen rejection.

Calcium signaling is known to regulate pollen hydration and tube growth through the modulation of vesicle trafficking and cytoskeletal organization [61,62,63]. In this study, Ca^2+^-responsive genes, including members of the CML and CPK families, showed sustained and elevated expression in self-pollinated stigma tissues. Notably, these genes have been previously associated with tip growth processes such as pollen tube elongation and root hair development [64,65]. Their persistent activation in SI conditions suggests that the dysregulation or overactivation of these pathways may interfere with normal pollen tube growth, potentially contributing to pollen rejection. In parallel, genes involved in actin dynamics, such as ADFs, exhibited similar expression patterns, further supporting the coordinated regulation of calcium signaling and cytoskeleton remodeling during the SI response.

ROS signaling also emerged as a key component of the SI response. In the stigma, the expression patterns of *Rboh* genes suggest that NADPH oxidase may contribute to ROS production, consistent with observations in the *Brassicaceae* [30]. In contrast, in the style, the enrichment of thylakoid-, photosystem-, and photosynthesis-related terms, together with the upregulation of ROS-scavenging enzymes such as SODs and GPXLs, suggests a possible association between ROS-related processes and plastid-associated metabolic responses. This apparent tissue-specific pattern may indicate that the stigma and style exhibit distinct transcriptional responses during SI, with the stigma acting as the primary recognition site and the style potentially participating in downstream or secondary responses rather than primary recognition. However, this pattern should be interpreted with caution, because style tissues, particularly the deeper layers, are not typically photosynthetically active. Therefore, the enrichment of chloroplast-related terms may reflect broader oxidative stress responses, plastid functions unrelated to active photosynthesis, such as leucoplast-associated metabolism, or signals from plastid-containing vascular or supporting tissues, rather than direct evidence for chloroplast-derived ROS production.

In the gametophytic self-incompatibility (GSI) systems of *Rosaceae* and *Solanaceae*, S-RNase secreted by stylar transmitting tract cells serves as the core executing factor [6,66]. Upon pollen tube entry into the style, stylar RNase (S-RNase) translocates into the pollen tube cytoplasm through apical membrane channels. When the S-genotype of the pollen matches that of the style-expressed S-RNase, the enzyme exerts specific ribonuclease activity, degrading intracellular RNA, disrupting metabolic pathways and cytoskeletal integrity, and ultimately inducing pollen tube growth arrest or programmed cell death. Recently [67], further showed that the S-RNase/SFB-mediated GSI system in *Prunus* is associated not only with S-locus specificity components, but also with SCF-related ubiquitin–proteasome regulation, auxin and ethylene signaling, calcium signaling, apoptosis, and defense-related pathways. Although yellow passion fruit does not appear to follow a typical S-RNase-based GSI mechanism, several downstream pathways identified in our study, including calcium signaling, hormone signaling, ROS/defense responses, and pollen tube growth-related processes, overlap with those reported in *Prunus* GSI. These similarities suggest that different SI systems may share partially conserved downstream regulatory modules, while their upstream recognition mechanisms remain distinct. Further evidence supporting divergence from classical SSI systems was provided by the expression patterns of key regulatory genes. Homologs of *Brassicaceae* SI components, such as FERONIA, MLPK, and ROP, exhibited elevated expression in style tissues rather than being restricted to the stigma. However, it should be emphasized that this observation is correlative and does not demonstrate a direct role in pollen recognition. The increased expression in the style may reflect downstream responses to pollen tube inhibition or general stress signaling, rather than indicating ectopic recognition events. In addition, the candidate SRK-like gene identified in this study showed substantial phylogenetic divergence from canonical SRK homologs, suggesting that the molecular basis of SI in passion fruit may differ from the classical SCR/SRK system, although functional validation is required.

Taken together, our results support a working hypothesis in which yellow passion fruit employs an SSI-like regulatory framework that incorporates conserved signaling modules, including calcium signaling, ROS, and cytoskeleton dynamics, but with altered spatial deployment and regulatory context. This may represent a lineage-specific adaptation of SI mechanisms, in which conserved components are reorganized across tissues to achieve pollen rejection. However, it is important to note that these conclusions are primarily based on transcriptomic evidence, and further genetic and functional studies will be required to validate the roles of candidate genes and to elucidate the precise molecular mechanisms underlying SI in passion fruit. In addition, it should be considered that the transcriptional signals observed in the style may represent a downstream consequence of pollen tube growth arrest, rather than a primary cause of SI recognition. The elevated expression of candidate genes in style tissues could reflect secondary responses associated with stress, defense activation, or metabolic adjustment following incompatible pollination. Another limitation of this study is the sampling time window. Because early pollen–stigma recognition events may occur rapidly and transiently, some primary SI signals may not have been fully captured, whereas transcriptional changes detected at later time points may partly reflect downstream signal amplification, stress responses, or consequences of pollen tube growth arrest. Therefore, distinguishing between causal regulatory mechanisms and consequential responses will be critical for future studies.

## 4. Materials and Methods

### 4.1. Plant Materials and Growth Conditions

All passion fruit plants (‘Baxi’ and ‘TN-1’) used in this study were obtained from the passion fruit (*Passiflora* spp.) germplasm conservation nursery at the Horticulture Research Institute, Guangxi Academy of Agricultural Sciences (Nanning, China). These plants were planted in February 2023, and the samples were collected during the peak flowering period of passion fruit in September 2023, when the material plants were 7 months old. Passion fruit plants were cultivated in a greenhouse under controlled conditions (16 h light/8 h dark; 200 μmol m^−2^ s^−1^ light intensity; 30–50% relative humidity; 28 ± 2 °C). Artificial pollination was carried out between 11:00 a.m. and 2:00 p.m. during the peak flowering period of the passion fruit. For cross-pollination (CP), the exposed white flower buds of ‘Baxi’ were selected, their stamens were removed with sterile tweezers before 10:00 a.m. and then the flowers were covered with breathable plastic bags. The matured ‘TN-1’ anthers were gently removed by using sterile forceps, and transferred into a 15 mL RNase-free centrifuge tube. During the peak flowering period of the ‘Baxi’ passion fruit, a sterile brush was used to transfer the ‘TN-1’ pollen onto the stigma of ‘Baxi’. For self-pollination (SP), the normally blooming ‘Baxi’ flower were chosen and covered with breathable plastic bags to prevent vector pollination. After the anthers had opened, a sterile brush was used to directly transfer the pollen of the same flower to its own stigma. For cut-stigma self-pollination (CSP), the exposed white flower buds of ‘Baxi’ were selected and the sterile tweezers were used to remove the stigma; the following steps were similar to those for SP.

### 4.2. Stigma Treatment and Pollen Tube Visualization

Passion fruit flowers at the early flowering stage, with undehisced anthers, were selected and emasculated. For the cut-stigma treatment, stigma papilla cells were carefully removed. All flowers were then manually pollinated with similar amounts of either self-pollen or cross-pollen. Entire pistils, including ovaries, were collected at 1, 4, 8, and 18 h after pollination and fixed in FAA solution (50% ethanol:glacial acetic acid:formaldehyde = 89:6:5). After fixation for 24 h, the samples were dehydrated through a graded ethanol series of 70%, 50%, and 30%, with approximately 10 min at each concentration, and then rinsed two to three times with distilled water. The samples were softened in 10 M NaOH at 56 °C in a water bath for 5–8 min, rinsed several times with distilled water, and stained with 0.1% aniline blue, optionally overnight. Finally, pollen attachment and germination on the stigma, as well as pollen tube elongation, were observed and imaged using a laser scanning confocal microscope ZEISS LSM 800 (Carl Zeiss AG, Oberkochen, Germany).

### 4.3. RNA Extraction and cDNA Library Construction

Stigmas and styles were collected before pollination and at different time points (0, 5, 15, and 30 MAP) after cross-pollination and self-pollination by using RNase-free forceps and scalpel blades. These samples were immediately frozen in liquid nitrogen and stored at −80 °C until RNA extraction. Three biological replicates were prepared for each sample. Total RNA was isolated from each sample (three biological replicates per condition) using the HiPure Plant RNAMini Kit (Magen Biotechnology, Guangzhou, China) according to the manufacturer’s instructions. For library construction, mRNA was enriched using oligo (dT) magnetic beads and fragmented into short segments. First-strand cDNA was synthesized with random hexamers, followed by second-strand synthesis using DNA polymerase I and RNase H. The double-stranded cDNA was then end-repaired, A-tailed, and ligated to adapters. Following size selection with AMPure XP beads (Beckman Coulter, Brea, CA, USA), the libraries were amplified by PCR and purified. Library quality was assessed by measuring concentration (Qubit 2.0), insert size (Agilent 2100, Agilent Technologies, Santa Clara, CA, USA), and effective concentration (Q-PCR). Finally, the libraries were sequenced on the DNBSEQ-T7 platform (DNBSEQ^TM^ Technologies, Shenzhen, China) using a paired-end 150 bp short-read sequencing strategy.

### 4.4. Differential Gene Expression Analysis

Raw reads were first processed with fastp [68] with default parameters to remove adapter sequences and low-quality reads, thereby obtaining high-quality clean reads for downstream analysis. The complete genome sequence of *P. edulis* was generated by our research group. The high-quality reads were subsequently aligned to the *P. edulis* reference genome using STAR v2.7.11b [69] with default settings. Gene abundance was quantified with Stringtie v3.0.0 [70] and normalized as transcripts per million (TPM). Differential expression analysis was performed using EdgeR v4 [71]. Genes with an absolute log_2_ fold change ≥ 1 and *p*-value < 0.05 were identified as differentially expressed genes (DEGs). Finally, functional enrichment analyses of gene ontology (GO) and Kyoto Encyclopedia of Genes and Genomes (KEGG) pathways were performed using the ClusterProfiler package v4.10.0 [72].

### 4.5. Staining of ROS

For stigmatic ROS staining, flowers at the initial opening stage before anther dehiscence were collected. Healthy flowers were selected, and their stamens were removed. Two pollination methods, self-pollination (SP) and cross-pollination (CP), were performed. Artificial pollination was then carried out using self-pollen or cross-pollen. Stigmas were excised at different time points after pollination (0, 5, 10, 15, 30, and 60 min) and immersed in MES-KCl buffer (MES 10 mM, KCl 5 μM, CaCl_2_ 50 μM, pH 6.15) for 30 min, followed by staining with 50 μM 2′,7′-dichlorodihydrofluorescein diacetate (H_2_DCFDA) for 1–2 h. After staining, the samples were washed at least three times and observed under a fluorescence microscope Olympus BX53 (Olympus Corporation, Tokyo, Japan).

## 5. Conclusions

In summary, our study provides a comprehensive transcriptomic analysis of the SI response in yellow passion fruit. Phenotypic observations confirm that the stigma serves as the primary site of pollen rejection, while transcriptional profiling reveals distinct temporal dynamics between compatible and incompatible pollination. Key SI-associated pathways, including calcium signaling, actin cytoskeleton dynamics, pectin modification, and ROS production, are activated following self-pollination. Notably, ROS appears to be generated through tissue-specific mechanisms: NADPH oxidase-mediated production in the stigma and chloroplast-derived production in the style. The expression patterns of conserved SI regulators, combined with the identification of a phylogenetically distinct SRK candidate, suggest that yellow passion fruit may employ a modified SSI mechanism. These findings provide a valuable transcriptomic framework for understanding SI in *Passiflora edulis* f. *flavicarpa* and offer insights that may ultimately facilitate breeding efforts in this economically important crop. Future functional studies, including genetic transformation and protein interaction analyses, will be essential to validate the roles of candidate genes identified in this study and to fully elucidate the molecular mechanism of SI in passion fruit.

## Data Availability

The genome assembly and annotation files reported in this study have been deposited in Figshare and are publicly available at https://doi.org/10.6084/m9.figshare.32144893. The raw transcriptome sequencing data used in this study have been deposited in CNGBdb under the accession number CNP0009450. The datasets used and/or analyzed during the current study are available from the corresponding author upon reasonable request.

## Supplementary Materials

The following supporting information can be downloaded at: https://www.mdpi.com/article/10.3390/plants15101564/s1, Figure S1: The phylogenetics tree of SRK genes; Figure S2: The expression profile of Rboh genes; Table S1: The gene ontology (GO) enrichment results of CP, SP groups at 5, 15, 30 MAP; Table S2: The expression profile of self-incompatibility (SI) associated genes; Table S3: The candidate transcription factors (TFs) involved in SI response.
