# Supplementary figures and images for: Deciphering the Transcriptomic Dynamics of Self-Incompatibility in Yellow Passion Fruit: Evidence of Modified Sporophytic Mechanism
Source: Plants (Basel). 2026 May 20;15(10):1564. doi: 10.3390/plants15101564 (PMC13210847; doi:10.3390/plants15101564)

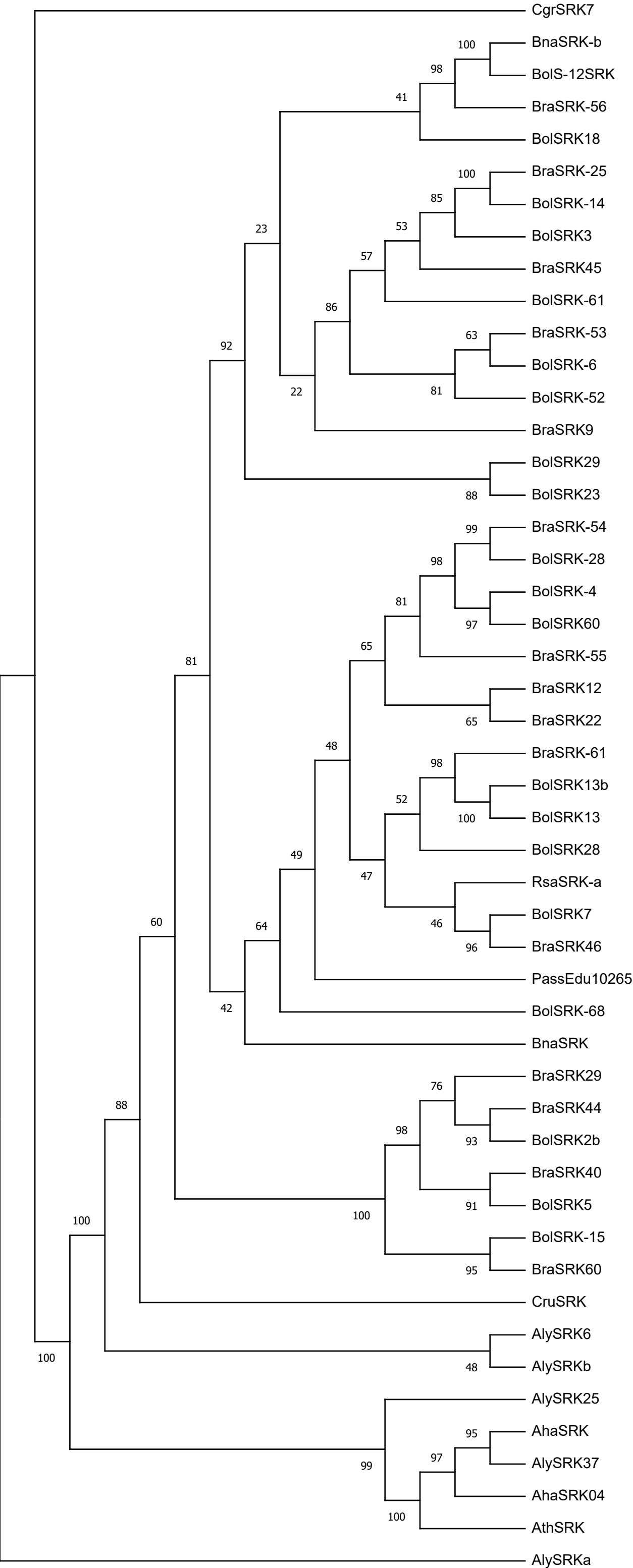

Supplement: Supplementary file 1 [file plants-15-01564-s001.zip › Figure S1.pdf]

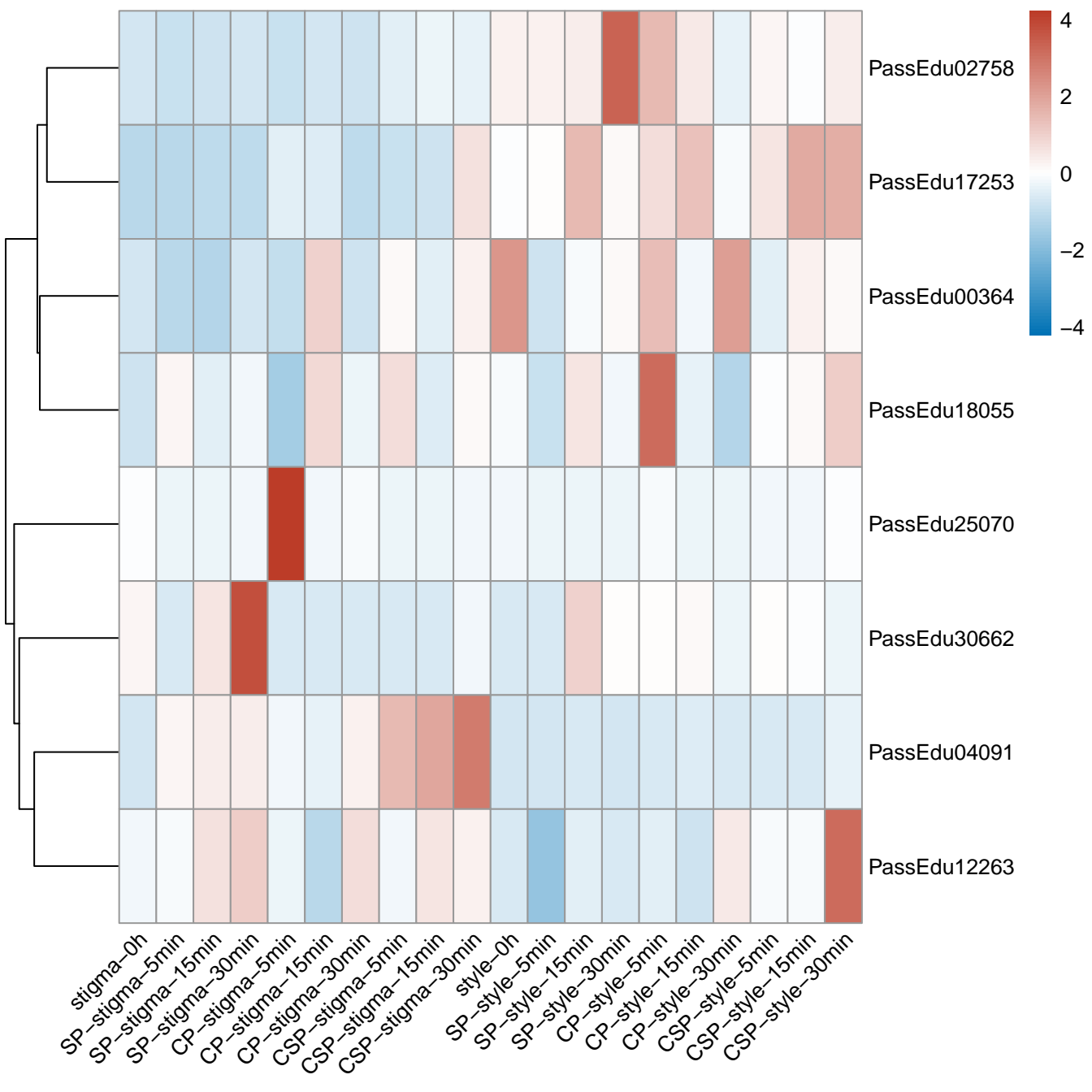

Supplement: Supplementary file 1 [file plants-15-01564-s001.zip › Figure S2.pdf]
